# Immune response to hepatitis B vaccination in HIV-positive individuals with isolated antibodies against hepatitis B core antigen: Results of a prospective Italian study

**DOI:** 10.1371/journal.pone.0184128

**Published:** 2017-09-01

**Authors:** Giulia Morsica, Sabrina Bagaglio, Vincenzo Spagnuolo, Antonella Castagna, Clelia Di Serio, Andrea Galli, Liviana Della Torre, Andrea Andolina, Alexander Pramov, Caterina Uberti-Foppa

**Affiliations:** 1 Division of Infectious Diseases, IRCCS, Ospedale San Raffaele, Milan, Italy; 2 Vita-Salute San Raffaele University, Milan, Italy; 3 Vita-Salute San Raffaele University, CUSSB (University Centre for Statistics in the Biomedical Sciences), Milan, Italy; Centre de Recherche en Cancerologie de Lyon, FRANCE

## Abstract

**Background and aim:**

Antibodies against hepatitis B core antigen (anti-HBc) are found in 14–44% of patients with HIV infection, but it is still unclear whether hepatitis B virus (HBV) vaccination should be recommended for HIV-positive subjects with isolated anti-HBc (IAHBc). We examined the rate of anamnestic and primary responses (ARs and PRs) and associated factors in a group of HIV-infected patients with an IAHBc profile.

**Methods:**

This prospective study recruited 25 HIV-positive patients with anti-HBc alone who were vaccinated against HBV infection. Those without an AR (anti-hepatitis B envelope antigen [anti-HBs] levels of <10 U/L) or who were hypo-responsiveness (anti-HBs levels of >10 but <100 U/L) four weeks after the first dose of vaccine underwent a full course of vaccinations. Their clinical and virological data, including the presence of occult hepatitis B infection (OBI), were evaluated in accordance with the vaccination schedule.

**Results:**

Six of the 25 patients (24%) showed an AR, four of whom had anti-HBs levels of <100 U/L. Ten of 19 (52.6%) remaining patients became seroprotected after the third dose. OBI was detected in four of the six patients with an AR, two of the 10 patients with a PR, and none of the nine patients who did not respond. Multivariate analysis showed that an AR was associated with the presence of OBI (P = 0.0162), and a PR was associated with HCV antibody status. (P = 0.0191).

**Conclusions:**

Our data suggest that testing for anti-HBc alone may not be a reliable means of assessing protection from HBV infection in HIV-positive patients. OBI-positive patients may benefit from a single vaccine dose. Anti-HCV serostatus may affect PRs.

## Introduction

Isolated anti-HBc (IAHBc) refers to a serological pattern in which antibodies against hepatitis B core antigen (anti-HBc) are present in the absence of hepatitis B surface antigen (HBsAg) or antibodies against HBsAg (anti-HBs). Among HIV-positive subjects, an IAHBc profile is found in 14–44% of patients [1–3], and is the most frequent serological pattern in patients with occult hepatitis B infection (OBI), defined as a positive serum polymerase chain reaction (PCR) assay for hepatitis B virus (HBV) DNA in the absence of HBsAg [4,5]. IAHBc may be encountered in subjects who have cleared HBV (in whom anti-HBs is lost or at undetectable levels) or as a false-positive result. If anti-HBs has never been formed or is truly lost, reinfection with HBV is possible [6]. The vaccination of these patients against HBV would elicit a primary anti-HBs response (an anti-HBs titre of <10 U/L) after a single vaccine dose, and an anti-HBs titre of >10 U/L after a full course of vaccinations. If immunological memory is preserved, re-exposure to HBV or a single vaccine dose would trigger a secondary or anamnestic anti-HBs response, defined as an anti-HBs titre >10 U/L [7].

Unlike the >90% response rates of the general population to standard HBV vaccination, HIV-infected patients show a low rate of response (37–85%) [8–11], but little is known [12–14] about their humoral immune response to vaccination if they have IAHBc. It is therefore unclear whether HBV vaccination should be recommended for immunocompromised subjects with an IAHBc profile, particularly if they are HIV positive. Furthermore, nothing is known about the possible effect of OBI on the humoral immune response during anti-HBV vaccination.

We prospectively evaluated the frequency of anamnestic and primary immune responses, and clinical or virological factors possibly involved in the response rate, in a group of HIV-infected patients an IAHBc profile.

## Materials and methods

### Patient selection and study design

In this prospective study, HBV vaccine (HBVAXPRO, 10 μg, Sanofi Pasteur MSD, Lyon, France) was offered to 25 HIV-infected patients who were consecutively recruited from among the outpatients attending the Department of Infectious Diseases, San Raffaele Hospital, Milan, Italy, on the basis of their anti-HBc positivity as the only marker of HBV infection one year before study entry. The anti-HBc serology of all 25 patients was confirmed before the vaccine was administered. The clinical and laboratory data used in the patient selection process were retrieved from an internal database (IDD-OSR database), and the patients were recruited between February and May 2014.

**Fig 1:**
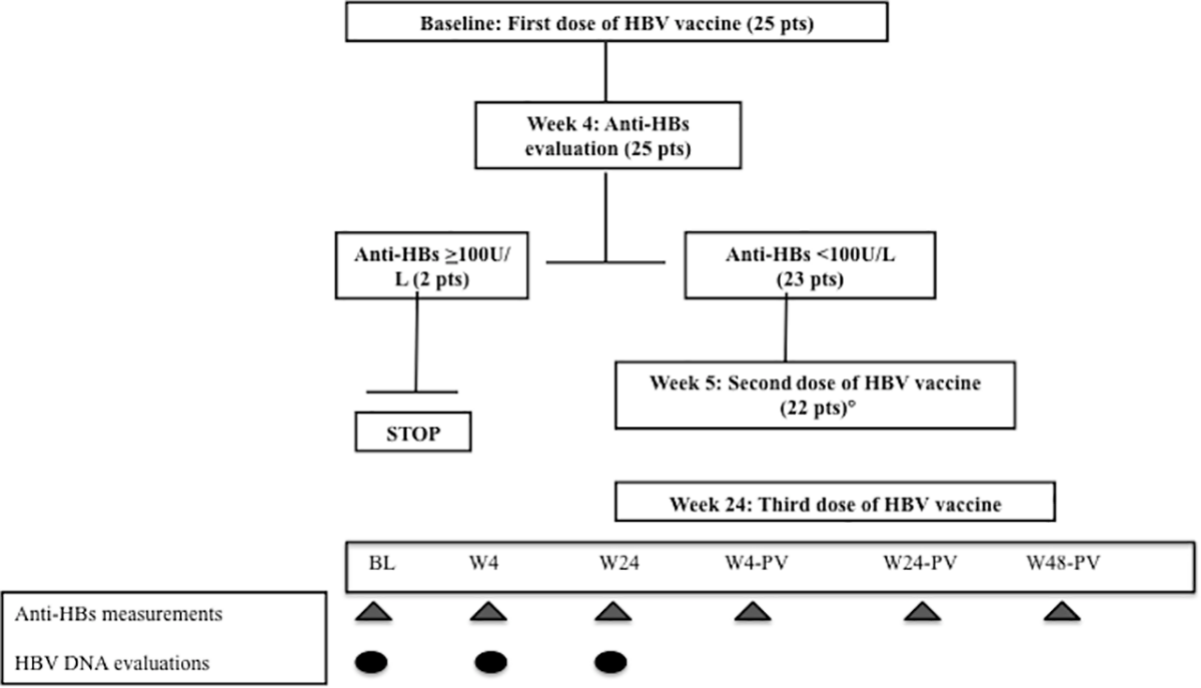
Schematic diagram of HBV vaccinations, anti-HBs measurements, and plasma sample collections for HBV DNA detection. °One patient was lost to follow-up after W4 and did not receive the full vaccination course.

The vaccine was administered at baseline (BL), in week 5 (W5) and in W24; the one-week delay in administering the second dose was necessary in order to check for an AR. Antibodies against the envelope S antigen were measured at BL, in W4 and W24, and then four and 24 weeks after the end of the full course of vaccinations (W4 post-vaccination [W4-PV] and W24-PV). An additional time point (W48-PV) after the third vaccine dose was considered. The patients without an AR (anti-HBs <10 U/L) or who were hyporesponsive (anti-HBs >10 U/L but <100 U/L) four weeks after the first dose underwent a full course of vaccinations. It was arbitrarily decided to give a full course of vaccinations to the hyporesponsive patients because asymptomatic HBV infection has been found in some healthy subjects with a poor response (anti-HBs 1–99 U/L,) [15,16] but not in those with titres of >100 U/L [17–19]. One patient who was hyporesponsive to the first dose did not complete the full course because he was lost to follow-up during the study period. The study design is summarised in Fig 1.

The study was conducted after being approved by the Ethics Committee of San Raffaele Hospital (Protocol No. FP13MORSICA).

### Laboratory methods

The quantitative assay of anti-HBs, and the qualitative assays of HBsAg, total immunoglobulin anti-HBc, anti-hepatitis B e antigen (anti-HBe), anti-HCV and anti-HIV were carried out using commercial enzyme immunoassay kits with a lower detection limit of 10 U/L (Architect, Abbott Diagnostics, Rome, Italy). HCV RNA, HIV RNA, and HBV DNA loads were quantified by means of routine laboratory assays (real-time PCR, Abbot Molecular, Rome, Italy): the detection limits of the assays are 12 IU/mL for HCV RNA, 40 copies/mL for HIV RNA, and 10 IU/mL (corresponding to 50 copies/mL) for HBV DNA.

#### Detection of occult HBV infection by means of highly sensitive polymerase chain reaction

The presence of OBI was evaluated at various times (see Fig 1) using a highly sensitive in-house nested PCR. Briefly, all of the DNA extracts were analysed for the presence of the HBV genome by means of different assays [20] using primers spanning the partial S (outers: sense 637–654, antisense 844–824; inners: sense 658–677, antisense 815–796), pre-C/C (outers: sense 1584–1604, antisense 2123–2104; inners: sense 1650–1669, antisense 1992–1973) and X regions (outers: sense 1266–1286, antisense1628–1608; inners: sense 1380–1400, antisense 1545–1520). The sensitivity limit is 105 pg/mL of cloned HBV DNA (2.6 copies/mL) [20]. We only considered the cases positive for at least two different viral genomic sequences at one or more of the three time points as having HBV DNA.

### Statistical analysis

#### Univariate analysis

The continuous variables are expressed as median values and interquartile ranges (IQRs), and the categorical variables as absolute counts and percentages (%). The data were analysed using Fisher’s exact test (for frequencies), or the Mann-Whitney U test (for continuous variables); a p-value of <0.05 was considered statistically significant.

#### Multivariate analysis

The aim of the multivariate analysis was to determine which covariates were most predictive of the outcome a AR *vs* no AR, and PR *vs* no response (NR). To this end, we used a multivariate logistic regression model aimed at predicting the binary response to treatment at BL considering the covariates listed in Table 1. The covariates included in the final model were selected on the basis of a systematic, stepwise model-selection procedure using the Akaike information criterion (AIC)[21], which makes it possible to find a model that is both parsimonious and capable of predicting the binary outcome. A p-value of <0.05 associated with the regression coefficients indicates statistical significance. Further details concerning the variable selection procedure can be found in the supporting information (S1 Table and S2 Table).

**Table 1:** Baseline characteristics of IAHBc/HIV-infected patients.

Patients	n = 25
**Males/females**	20/5
**Age, years**	50 (45–54)
**Duration of HIV infection, years**	17 (6–28)
**Duration of ART, years**	14 (4–19)
**Risk factors** **Sexual exposure / IVDU#**	12/13
**CD4**^**+**^ **count, cells/mm**^**3**^	588 (430–746)
**CD8**^**+**^ **count, cells/mm**^**3**^	784 (610–1144)
**CD4**^**+**^**/CD8**^**+**^ **ratio**	0.74 (0.48–0.89)
**Nadir CD4**^**+**^ **count, cells/mm**^**3**^	209 (147–279)
**Nadir CD4**^**+**^**/CD8**^**+**^ **ratio**	0.20 (0.13–0.28)
**AST (U/L)°**	27 (22–47)
**ALT (U/L)°°**	35 (24–54)
**Anti-HCV (pos/neg)**	14/11
**HCV RNA Log IU/mL** **(No. patients = 14)**	5.56 (1.89–6.33)

Median, first and third quartile (in brackets) are reported for continuous variables. Absolute counts are reported for nominal variables. #IVDU = intra-venous drug users.

°AST = aspartate amino transferase levels, (normal value <35U/L)

°°ALT = alanine amino transferase levels (normal value<41U/L).

## Results

### Baseline characteristics

Table 1 shows the general characteristics of the IAHBc/HIV-infected patients, who were prevalently males. Fourteen (56%) of the patients had antibodies against HCV (anti-HCV), 12 (92.3%) of whom were intravenous drug users (IVDUs) and two (16.7%) were exposed to the risk of sexual transmission. HCV RNA was detectable in ten of the 14 (71.4%) anti-HCV positive patients.

All of the patients were receiving anti-retroviral therapy (ART), showed a good immunovirological response, and had undetectable HIV RNA loads (<40 copies/mL). Thirteen patients were receiving ART with anti-HBV activity. Routine laboratory assays showed that all of the patients were anti-HBe negative and HBV DNA, which was tested for at least at twice (BL and W4 in all patients, and also at W24 in 21 patients) was invariably undetectable (<10 IU/mL).

### Humoral immune response to vaccination

Six of the 25 patients (24%) showed an AR (anti-HBs >10 U/L) after the first vaccine dose (W4) and, in accordance with the study protocol, the two who had an anti-HBs titre of >100 U/L did not undergo the full course of vaccinations. Ten of the 19 remaining patients (52.6%) showed a PR (anti-HBs >10 U/L) after a full course of vaccinations.

### Detection of occult HBV infection

Our highly sensitive homemade nested PCR aimed at revealing the presence of OBI. HBV DNA was intermittently detected in six of the 16 (37.5%) patients with an AR or PR and in none of the nine unresponsive patients.

### Predictors of an AR to HBV vaccination at univariate and multivariate analysis

The univariate analysis, which separately tested the effect of the covariates on AR *vs* no AR, showed that the presence of OBI was significantly associated with response (P = 0.015); while the other analysed variables were not (Table 2).

**Table 2:** Characteristics of patients with and without an anamnestic response (AR).

	AR n = 6	No AR n = 19	P-value
**Males/females**	5/1	15/4	1.000
**Age (years)**	50(49–50)	51 (45–54)	0.655
**Duration of HIV infection (years)**	25 (7–28)	13 (9–28)	1.000
**Duration of ART (years)**	18 (6–19)	13 (4–18)	0.701
**Risk factors, sexual exposure / IVDU#**	2/4	10/9	0.645
**CD4**^**+**^ **count (cells/mm**^**3**^**)**	484 (436–526)	636 (437–801)	0.138
**CD8**^**+**^ **count (cells/mm**^**3**^**)**	853 (707–1082)	784 (501–1151)	0.707
**CD4**^**+**^**/CD8**^**+**^ **ratio (cells/mm**^**3**^**)**	0.57 (0.36–0.77)	0.74 (0.53–0.94)	0.141
**Nadir CD4**^**+**^ **count (cells/mm**^**3**^**)**	155 (132–164)	236 (178–287)	*0*.*059*
**Nadir CD4**^**+**^**/CD8**^**+**^ **ratio (cells/mm**^**3**^**)**	0.16 (0.11–0.19)	0.23 (0.19–0.37)	*0*.*091*
**AST (U/L)°**	23 (19–34)	28 (25–53)	0.06
**ALT (U/L)°°**	21 (17–34)	39 (27–70)	*0*.*056*
**Anti-HCV (pos/neg)**	4/2	10/9	0.661
**HCV RNA (Log IU/mL)** **(No. patients = 14)**	1.04 (1.04–2.55)	6.23 (4.9–6.33)	0.252
**OBI**	4	2	**0.015**

Median, first and third quartile (in brackets) are reported for continuous variables. Absolute counts are reported for nominal variables. #IVDU = intra-venous drug users.

°AST = aspartate amino transferase levels, (normal value <35U/L)

°°ALT = alanine amino transferase levels (normal value<41U/L).

In the subsequent multivariate logistic regression analysis using a stepwise selection procedure, the probability of obtaining an AR in the presence of OBI significantly increased (coefficient 4.37, P = 0.016), whereas the coefficient of baseline CD4+ T cell levels was -0.007227 (P = 0.0756). The detailed estimates can be found in the supporting information (S1 Table).

### Predictors of a PR to HBV vaccination at univariate and multivariate analysis

The univariate analysis, which separately tested the effect of the covariates on PR *vs* NR, showed that the duration of HIV infection (P = 0.044) and anti-HCV status (P = 0.023) were significantly different in the two groups (Table 3).

**Table 3:** Characteristics of patients with a primary response (PR) or no response.

	PR n = 10	No response n = 9	P
**Males/females**	6/4	9/0	0.087
**Age (years)**	53 (49–54)	50 (44–54)	0.566
**Duration of HIV infection (years)**	27 (15–30)	12 (6–13)	**0.044**
**Duration of ART (years)**	17 (13–21)	5 (2–13)	*0*.*071*
**Risk factors, Sexual exposure / IVDU#**	3/7	7/2	*0*.*070*
**CD4+ count (cells/mm**^**3**^**)**	709 (406–879)	605 (476–62)	0.720
**CD8+ count (cells/mm**^**3**^**)**	732 (495–834)	1136 (774–1260)	0.230
**CD4+/CD8+ ratio (cells/mm**^**3**^**)**	0.82 (0.64–1.12)	0.74 (0.50–0.86)	0.380
**Nadir CD4+ count (cells/mm**^**3**^**)**	223 (114–274)	260 (199–290)	0.447
**Nadir CD4+/CD8+ ratio (cells/mm**^**3**^**)**	0.23 (0.15–0.30)	0.24 (0.19–0.41)	0.713
**AST (U/L)°**	37 (27–49)	26 (22–59)	0.270
**ALT (U/L)°°**	45 (30–65)	35 (26–72)	0.744
**Anti-HCV (pos/neg)**	8/2	2/7	**0.023**
**HCV RNA (Log IU/mL)** **(No. patients = 10)**	5.56	6.31	**-**
**OBI**	2	0	0.473

Median, first and third quartile (in brackets) are reported for continuous variables. Absolute counts are reported for nominal variables. #IVDU = intra-venous drug users.

°AST = aspartate amino transferase levels, (normal value <35U/L)

°°ALT = alanine amino transferase levels (normal value<41U/L).

In the multivariate analysis made using the same stepwise selection procedure, the final model retained only anti-HCV positivity, which had a significant positive impact on the probability of obtaining a PR (coefficient 2.6391, P-value = 0.0191). The detailed estimates can be found in the supporting information (S2 Table).

### Anti-HBs persistence

Eighty-five percent (11/13) of the patients who underwent a full course of vaccinations were responders in W24-PV. Anti-HBs was measured again in 12 patients in W48-PV, and 41.6% (5/12) still had anti-HBs titre of >10 U/L. The two patients with an AR and anti-HBs titres of >100 U/L after the first dose of vaccine (W4), were still anti-HBs positive in W48-PV.

## Discussion

The significance of an isolated anti-HBc (IAHBc) serological pattern and the importance of vaccinating IAHBc-positive subjects in high-risk populations (particularly drug users) is still debated because there are few published data concerning HIV-positive patients with an IAHBc profile.

Twenty-four percent of our HIV/IAHBc-positive patients showed an AR to HBV vaccination after undergoing a standard dose vaccination schedule, which suggests that the majority of patients with an IAHBc profile are not protected. This is in line with studies showing AR rates of 7–32.5% of such patients [12–14], but the seroprotection rate obtained after one dose of vaccine is lower than 46% found in a very recent study [22]. However, the patients were younger than our patients, the duration of both HIV infection and ART was shorter, thus suggesting more recent HBV infection and a consequently greater probability of not having fully lost anti-HBs. It is therefore possible that the differences in patient characteristics contributed to this different result.

Unlike previous studies that have found an association between ARs and CD4+/CD8+ ratios [22] or the acquisition of HIV as a result of the use of injected drugs [14], our multivariate analysis showed that an AR was more likely in patients with OBI than in those without.

This is the first prospective study of the presence of OBI during an HBV vaccination programme using highly sensitive PCR assay. HBV vaccinations have previously been administered to HBc positive individuals with undetectable HBV-viraemia at baseline [12,22], but in these cases HBV DNA was measured just once in each patient, and by using routine laboratory assay, which may explain the conflicting results.

The current European guidelines for the treatment of chronic hepatitis B and C in HIV infected patients [23] do not recommend vaccinating of IAHBc–positive individuals and consequently do not provide information on the efficacy and the significance of vaccination in anti-HBc, OBI-positive patients, although some authors [24–26] have suggested determining HBV DNA levels in order to rule out OBI in individuals who fail to respond to HBV vaccination before giving a primary HBV immunisation series.

Our findings show that IAHBc and OBI-positive individuals should receive at least one vaccine dose, and that the vaccination is immunogenic in such patients.

There are contrasting data concerning the effect of HCV status on the immunogenicity of active vaccination against HBV: some studies indicate no correlation between anti-HCV serostatus and humoral response [13, 27–30] whereas others have found a lower response rate in anti-HCV positive individuals [12,31,32]. However, the majority of these studies involved HIV- negative individuals without any serological marker of HBV. Our multivariate analysis showed that HCV exposure is associated with the anti-HBs seroconversion rate in individuals achieving PR. It can be speculated that individuals with IAHBc and HCV infection have previously had a genuine HBV infection with undetectable anti-HBs levels due to immune dysfunction probably caused by concomitant HCV coinfection [33,34]. The recall provided by a single vaccine dose is probably not sufficient to elicit an AR in such patients, whereas the immunogenic stimulus offered by a further to doses may trigger a specific humoral response. We also evaluated the maintenance of anti-HBs of >10U/L, after a full course of vaccinations showing that 85% of our patients had anti-HBs titres of >10 U/L at W24-PV, a proportion that dramatically decreased to 41.6% at W48-PV.

The longevity of the humoral response was similar to that reported by Kaech *et al*., [14] who used a classical vaccination regimen, but was less than that described by Piroth *et al*. [22], who used a reinforced regimen. We therefore agree with the finding of Piroth *et al*. that a new vaccination strategy based on reinforced doses or adjuvant vaccine (thus extending the persistence of antigen) may lead to higher PR rates and longer immunogenicity. In conclusion, although we are aware that we analysed only a small group of patients, there is strong statistical support for the view that anti-HBc positivity alone is not a reliable marker of protection against HBV infection in HIV-positive individuals. OBI-positive individuals may benefit from a single dose of HBV vaccine, whereas anti-HCV serostatus seems to be associated with PRs.

Finally, anti-HBc-positive individuals with anti-HBs titres of >100 U/L four weeks after a single recall dose of HBV vaccine may not need further vaccination.

## Supporting information

S1 Table Analysis 1 (anamnestic response vs non-anamnestic response). Estimated coefficients and associated standard errors and p-values of a logistic regression for the binary outcome “anamnestic response vs no anamnestic response”. The estimated coefficients and associated standard errors are reported in log-odds scale.
(DOCX)

S2 Table Analysis 2 (primary response vs no-response). Estimated coefficients and associated standard errors and p-values of a logistic regression for the binary outcome “primary response vs no response”. The estimated coefficients and associated standard errors are reported in log-odds scale.
(DOCX)
