# Supplementary material for: Immune response to hepatitis B vaccination in HIV-positive individuals with isolated antibodies against hepatitis B core antigen: Results of a prospective Italian study
Source: PLoS One. 2017 Sep 1;12(9):e0184128. doi: 10.1371/journal.pone.0184128 (PMC5581175; doi:10.1371/journal.pone.0184128)
Supplement: S1 Table — Estimated coefficients and associated standard errors and p-values of a logistic regression for the binary outcome “anamnestic response vs no anamnestic response”. The estimated coefficients and associated standard errors are reported in log-odds scale. (DOCX) [file pone.0184128.s001.docx]

**Table S1. Analysis 1 (anamnestic response vs non-anamnestic response)**

Estimated coefficients and associated standard errors and p-values of a logistic regression for the binary outcome “anamnestic response vs no anamnestic response”. The estimated coefficients and associated standard errors are reported in log-odds scale.

|  | **Estimate** | **Std. Error** | **P value** |
| --- | --- | --- | --- |
| **Intercept** | 1.157684 | 1.710647 | 0.4986 |
| **OBI** | 4.370521 | 1.817044 | 0.0162 |
| **BL_CD4** | -0.007227 | 0.004068 | 0.0756 |
