# Supplementary material for: Immune response to hepatitis B vaccination in HIV-positive individuals with isolated antibodies against hepatitis B core antigen: Results of a prospective Italian study
Source: PLoS One. 2017 Sep 1;12(9):e0184128. doi: 10.1371/journal.pone.0184128 (PMC5581175; doi:10.1371/journal.pone.0184128)
Supplement: S2 Table — Estimated coefficients and associated standard errors and p-values of a logistic regression for the binary outcome “primary response vs no response”. The estimated coefficients and associated standard errors are reported in log-odds scale. (DOCX) [file pone.0184128.s002.docx]

**Table S2. Analysis 2 (primary response vs no-response)**

Estimated coefficients and associated standard errors and p-values of a logistic regression for the binary outcome “primary response vs no response”. The estimated coefficients and associated standard errors are reported in log-odds scale.

|  | **Estimate** | **Std. Error** | **P value** |
| --- | --- | --- | --- |
| **Intercept** | -1.2528 | 0.8018 | 0.1182 |
| **HCVAb1** | 2.6391 | 1.1260 | 0.0191 |
